# Infrarenal endovascular repair of abdominal aortic aneurysm with hostile aortic neck configuration by primary placement of an infrarenal Palmaz stent followed by an endoprosthesis with suprarenal fixation (the “Neoneck” technique)

**DOI:** 10.1590/1677-5449.202400452

**Published:** 2025-02-14

**Authors:** André Pinheiro Ribeiro Alves, Queise da Costa Cettolin, Yasmin Falcon Lacerda, Mauricio de Amorim Aquino, Gustavo dos Santos Domingues

**Affiliations:** 1 Hospital Geral Roberto Santos – HGRS, Salvador, BA, Brasil.; 2 Hospital Santa Izabel, Santa Casa de Misericórdia da Bahia, Salvador, BA, Brasil.

**Keywords:** endovascular aortic repair, infrarenal abdominal aortic aneurysm, tortuous neck, hostile neck, proximal neoneck, Palmaz stent, reparo aórtico endovascular, aneurisma de aorta abdominal infrarrenal, colo tortuoso, colo hostil, Neocolo proximal, Palmaz

## Abstract

**Background:**

The Endovascular Repair (EVAR) is the first-choice technique for Abdominal Aortic Aneurysm (AAA). Treatment success is dependent on favorable anatomy and an adequate sealing zone formed by a straight aortic neck (slightly angled). Endoprostheses implanted at critical aortic angles (above 75º) may result in unfavorable results such as fracture, migration, and type Ia endoleak. The technique for creating a proximal “Neoneck” consists of implanting the Palmaz stent in the proximal neck of the AAA, before placement of the endoprosthesis, allowing remodeling and rectification of the aortic neck.

**Objectives:**

To describe the “Neoneck” technique and report the early results of three cases with rectification of the proximal neck angle using a Palmaz stent, enabling treatment in these cases with angulated necks.

**Methods:**

We analyzed data collected from patients in whom Palmaz stents were placed, constructing a proximal Neoneck, during EVAR for infrarenal AAA with very tortuous proximal aortic neck, assessing anatomy, devices and perioperative results, including success rates, complications, mortality, and patency in the short and medium term.

**Results:**

All patients presented satisfactory evolution with immediate technical success. There were no cases of migration, fracture, or type Ia endoleaks. There was evidence of aneurysmal sac reduction after six months. There were no complications related to surgical access or deaths.

**Conclusions:**

In cases of angled aortic necks, when open AAA repair is not possible, in the absence of ideal devices or in urgent cases, prior rectification of the aortic neck deploying the Palmaz stent is feasible and effective. Long-term studies are still needed to validate the technique and assess safety.

## Resumo

**Contexto:**

O reparo endovascular do aneurisma de aorta abdominal infrarrenal (EVAR) representa hoje a técnica de escolha inicial. O sucesso terapêutico depende de anatomia favorável e adequada zona de selamento, formada por um colo reto (pouco angulado). Endopróteses implantadas em angulações críticas (acima de 75º) podem resultar em resultados desfavoráveis como fratura, migração e *endoleak* tipo 1A. A técnica de confecção de um “Neocolo” proximal consiste no implante do *stent* Palmaz no colo proximal do AAA de forma primária no mesmo tempo cirúrgico, antes da colocação da endoprótese, permitindo o remodelamento e a retificação do colo aórtico.

**Objetivos:**

Descrever a técnica de “Neocolo” e relatar resultados iniciais em três casos de EVAR com retificação da tortuosidade do colo proximal com o uso de *stent* Palmaz, viabilizando o tratamento nos casos de colos proximais muito angulados.

**Métodos:**

Dados foram coletados de pacientes submetidos à técnica de Neocolo durante EVAR para AAA infrarrenal com colo proximal, avaliando anatomia, dispositivos e resultados perioperatórios.

**Resultados:**

Todos os pacientes apresentaram evolução satisfatória com sucesso técnico imediato. Não houve casos de migração, fratura e/ou *endoleak* 1A. Houve evidência de redução do saco aneurismático após 6 meses. Não houve complicações relacionadas ao acesso cirúrgico e/ou óbitos.

**Conclusões:**

Em casos de colos muito tortuosos, na impossibilidade do reparo aberto do AAA, na ausência de dispositivos dedicados ou em casos de urgência, a retificação prévia do colo com o *stent* Palmaz é viável e eficaz. Estudos a longo prazo ainda são necessários para validação da técnica e avaliação da segurança.

## INTRODUCTION

Infrarenal Abdominal Aortic Aneurysm (AAA) occurs in approximately 4% to 8% of the population.^1-3^ Endovascular aneurysm repair (EVAR) is the gold standard treatment^4-6^ and its success is fundamentally dependent on favorable anatomy, with an adequate sealing zone, preferably formed by a straight aortic neck (slightly angled), of adequate length, without calcifications or thrombi.^4,7,8^ In many cases, however, severe angles are seen in the proximal neck, representing an important obstacle to a satisfactory outcome of the infrarenal EVAR for AAA.^8^

Endoprostheses placed at critical angles (greater than 75º) may result in unfavorable results such as fracture, migration, type Ia endoleak, and even aneurysm rupture. When available, fenestrated/customized endoprostheses can be deployed for visceral arteries, allowing sealing at a proximal region of the aorta in a better aligned area, with a higher success rate. Unfortunately, the high cost of these devices and the time required to manufacture them (minimum of 15 days) often make their use impractical, especially in urgent situations.^8^

One technique recently developed for very tortuous necks involves making a proximal “Neoneck”. This resource consists of using a balloon-expandable Palmaz stent (P4014 Cordis®) implanted at the proximal neck of the AAA during the same surgical intervention, before placement of the endoprosthesis, allowing remodeling and rectification of the aortic neck for the subsequent infrarenal EVAR, thus avoiding type Ia endoleak.^9^ The Palmaz stent is traditionally used to correct type Ia endoleaks in settings in which sealing is difficult, aiming to increase the radial force exerted by the prosthesis on the proximal neck and maximize its contact with the aortic wall.^10,11^ In the method described, however, the stent is deployed prior to endoprosthesis placement, straightening the aortic neck for a better fit and EVAR success.^9^

This study aims to describe the “Neoneck” technique and report the early results of three cases of infrarenal aortic aneurysms treated at a specialized center for treatment of aortic diseases in Salvador, Bahia, Brazil, with endovascular repair in conjunction with preparatory rectification of the tortuosity of the proximal aortic neck using a Palmaz stent, creating a straight Neoneck and enabling EVAR.

## METHODS

### Study design

A retrospective analysis was performed based on data collected from three patients in whom Palmaz stents were placed, constructing a proximal “Neoneck”, during EVAR for infrarenal AAA with very tortuous proximal aortic necks, from December 2021 to July 2023.

### Patients

Patients were included with aneurysmal dilatation of the infrarenal abdominal aorta (whether aneurysm or dilated dissection) who had a proximal neck with severe angulation (greater than 75º), in whom it was necessary to construct a new neck with a steel stent to allow adequate EVAR.

Data were collected and analyzed on demographics, anatomy (including extension, diameter, and angulation of aortic neck), devices deployed for treatment, besides perioperative results and treatment follow-up in acute and subacute settings, including immediate and late postoperative success rates, complications – in particular, endoleaks –, morbidity, mortality, and patency in the short and medium term.

All patients were treated at a single center, and the signed consent was obtained from all. This study was approved by the Research Ethics Committee at the HGRS (Ethics Appraisal Submission Certificate: 69734323.1.0000.5028, Consolidated Opinion: 6.574.411).

### The Neoneck technique

For treatment, we used the balloon-expandable Palmaz stent (P4014/Cordis®) mounted on Maxi LD/Cordis® or Atlas/Bard® balloon catheters, employing an average graft oversizing of 10 to 20% of the proximal neck diameter, followed by placement of suprarenal fixation endoprostheses, employing an oversizing of approximately 20% of the diameter of the proximal aortic neck.

**Figure 1:**
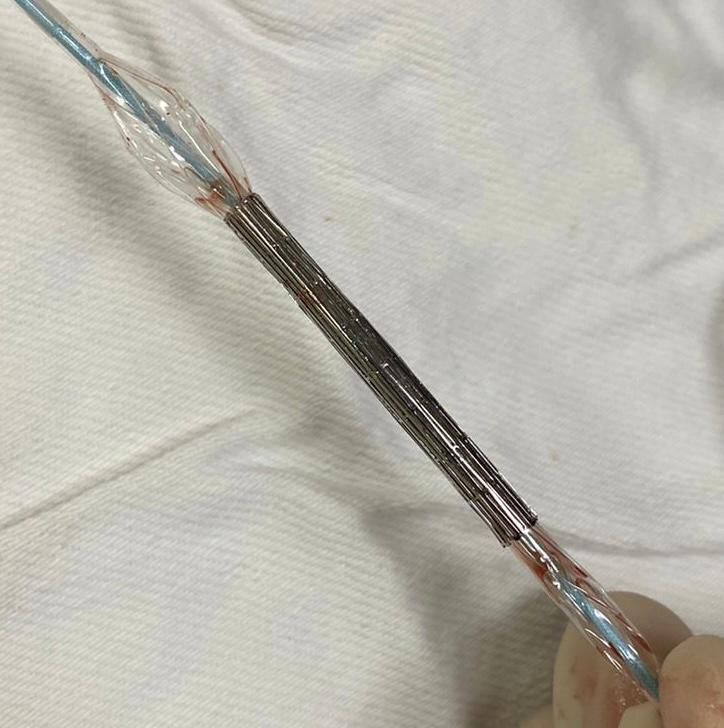
Image depicting the balloon partially inflated before passing through the femoral introducer sheath, creating resistance to distal migration of the Palmaz stent.

**Figure 2:**
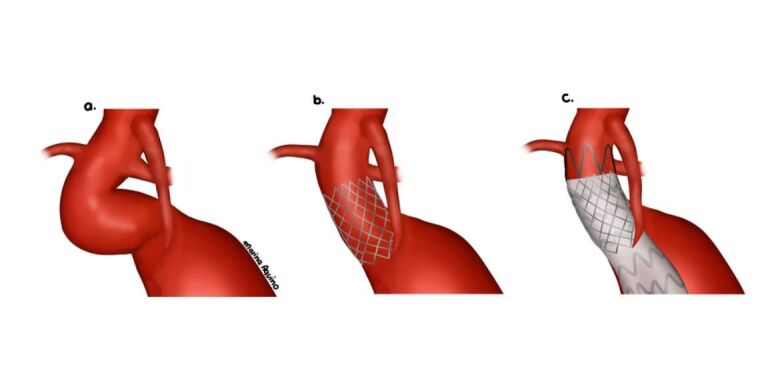
Schematic drawing demonstrating the technique for creating a Neoneck with a Palmaz stent prior to EVAR.

All patients underwent femoral access by surgical dissection. Initially, a 14F x 40cm sheath was placed in the common femoral artery. Subsequently, a 0.035 ” x 260cm extra-stiff guidewire was passed through the femoral access, followed by previous assembly of the Palmaz stent on a Maxi LD/Cordis® or Atlas/Bard® balloon catheter. To avoid migration of the stent over the balloon during passage through the femoral introducer valve, the balloon was slowly inflated outside the patient, creating resistance to distal migration of the stent (Figure 1). Then, positioning angiography was performed, and the Palmaz stent mounted on the balloon catheter was implanted in an infrarenal position (Figure 2). Finally, the standard technique for endovascular treatment of the AAA was performed, with placement of the bifurcated endoprosthesis.

Pre-treatment and control CT angiography (AngioCT) were performed in the bioimaging department of the HGRS, at intervals of 1, 3, and 6 months postoperative. The images were analyzed using Osirix software®.

### Definitions

Technical success is defined as evidence of total aneurysm exclusion on the control intraoperative angiography, without type Ia (proximal neck) or type III (material and device connections) endoleaks, and maintenance of patency of visceral arteries. Late postoperative success is defined as maintenance of these results on control CT scans after 1 and 6 months.

### Statistics

Continuous data were expressed as mean ± standard deviation or median and interquartile range, if the distribution was not normal. Categorical data were expressed as counts and percentages.

Considering the references in the literature, with a prevalence of 4-8% of Infrarenal Abdominal Aortic Aneurysms, of which approximately 48-53% have hostile necks, with a sampling error of 5% and a 95% confidence interval, an ideal sample size of 399 patients was found. This study describes the technique and the initial experience of the first three cases treated at the service, without a sufficient number for an ideal sample. The STROBE protocol was followed.

## RESULTS

Between December 2021 and July 2023, three patients with AAA with a very tortuous proximal aortic neck underwent EVAR using the Neoneck technique (Table 1).

**Table 1:** Demographic data, aortic pathology, anatomical characteristics, endoprosthesis devices used, stents, and types of balloons used in this study.

**ID**	**Sex/age (y)**	**Comorbidities**	**Aortic pathology**	**Proximal angle (º)**	**Neck diameter (mm)**	**Neck length (cm)**	**Endoprosthesis device**	***Neoneck* stent**	**Balloon catheter**
1	F/67y	SAH, CHF	Abdominal aortic aneurysm	105	20	5.6	Incraft/Cordis®	Palmaz (P4014/Cordis)	Maxi LD/Cordis®
2	F/62y	SAH, ovarian cancer	Dilated abdominal aortic dissection	91	17	3.4	E- tegra/Artvion®	Palmaz (P4014/Cordis)	Atlas/Bard®
3	F/94y	SAH	Abdominal aortic aneurysm	93	18	3.8	Incraft/Cordis®	Palmaz (P4014/Cordis)	Maxi LD/Cordis®

Key: F (female), y (years), SAH (systemic arterial hypertension), CHF (congestive heart failure).

Analyzing demographic data, in regards to sex, all patients were female. All patients had significant comorbidities and/or advanced age that restricted the choice of open repair due to unfavorable prognosis. Systemic arterial hypertension was the most prevalent pathology, present in all patients. Mean age was 74.3 years (62-94 years) and median age was 67 years.

Regarding aortic pathology and anatomy, two patients had AAA and one patient had an isolated dilated dissection of the abdominal aorta. The mean diameter of the aortic dilation was 6.3cm (4.8-8.0cm) and the median diameter was 6.1cm. The mean length of the proximal neck was 4.3cm (3.4-5.6cm) and the median length was 3.8cm. The mean angle was 96 degrees (91º-105º) and the median angle was 93º.

Regarding femoral access, all patients had femoral arteries with acceptable caliber (mean 8mm) and no significant calcifications.

**Figure 3:**
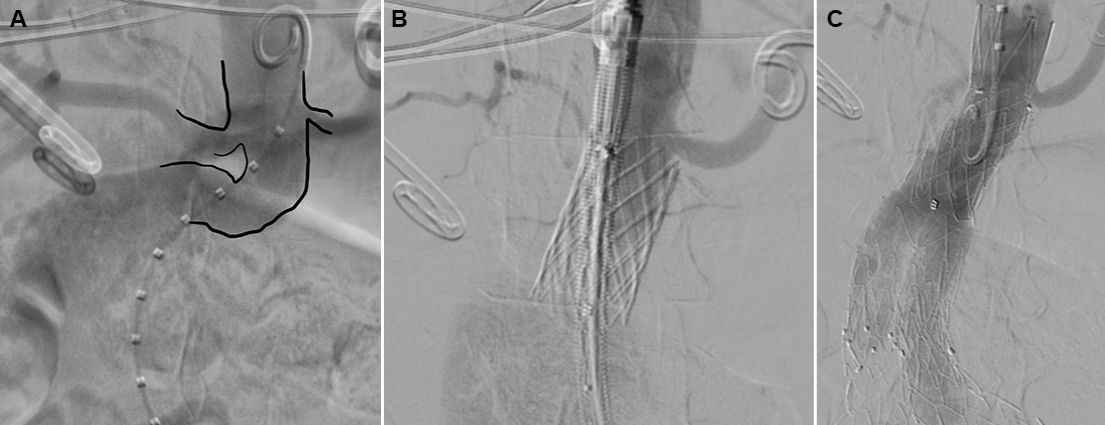
Intraoperative angiography images. **A)** First, showing a tortuous aortic neck. **B)** Second, primary Palmaz stent already released, before the endoprosthesis is deployed, showing rectification of the proximal neck. **C)** Third, Palmaz stent and endoprosthesis already placed, with no evidence of type Ia endoleak.

All procedures in this series were performed with technical success (Figure 3). Mean fluoroscopy time was 81 minutes (60-97 min). There were no cases of migration, fracture, or type IA endoleak. The average volume of iodinated contrast used was 120mL and none of the patients had renal dysfunction after surgery.

**Figure 4:**
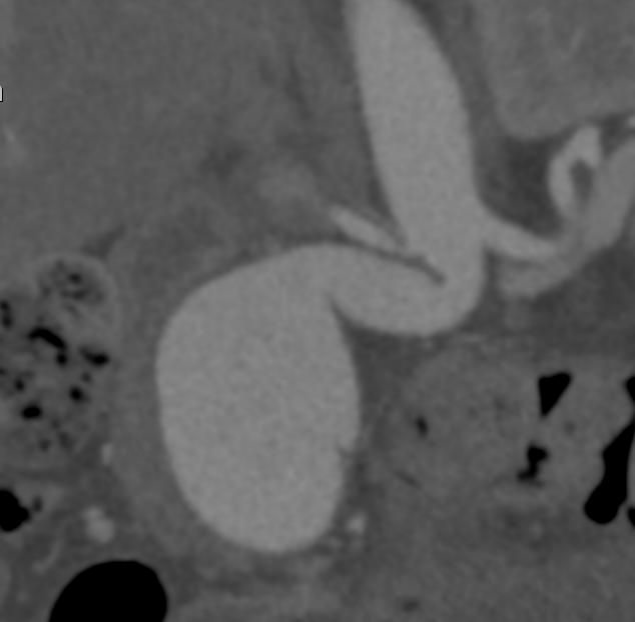
Preoperative CT angiography image showing tortuous aortic neck.

**Figure 5:**
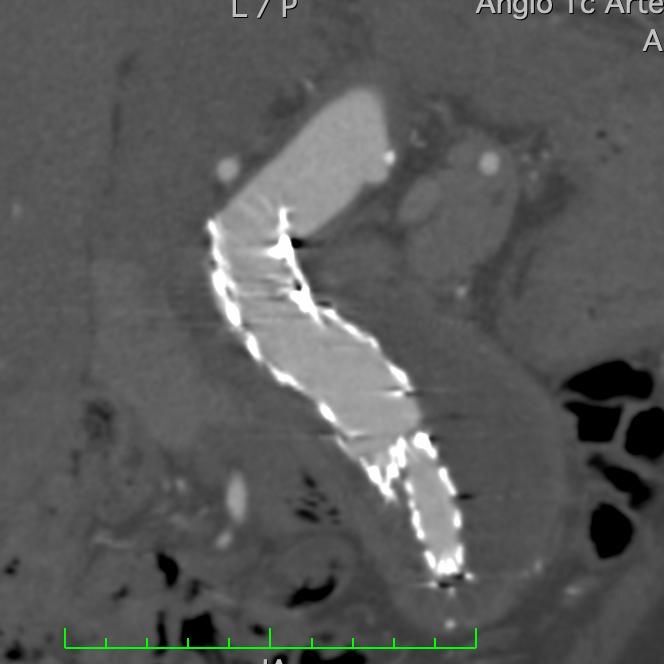
CT angiography image 30 days after EVAR with the Neoneck technique, showing rectification of the aortic neck, with no endoleaks.

**Figure 6:**
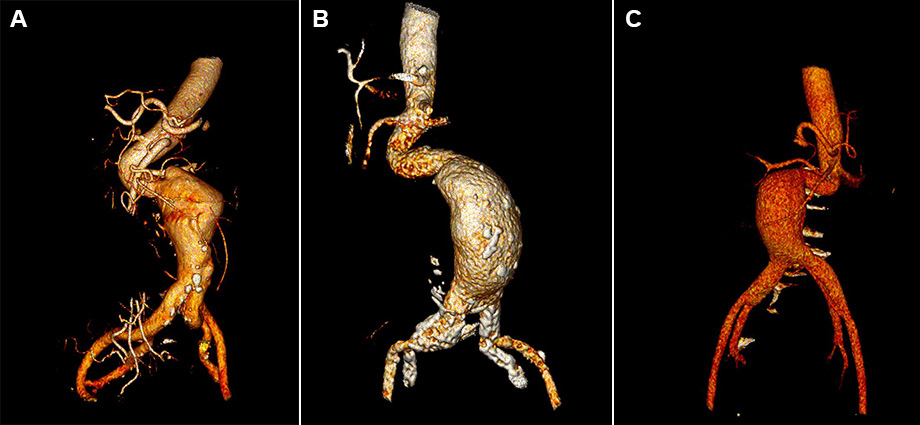
Preoperative 3D CT angiography reconstructions showing tortuous aortic neck. (A) 3D CT reconstruction of the first patient; (B) 3D CT reconstruction of the second patient; (C) 3D CT reconstruction of the third patient.

**Figure 7:**
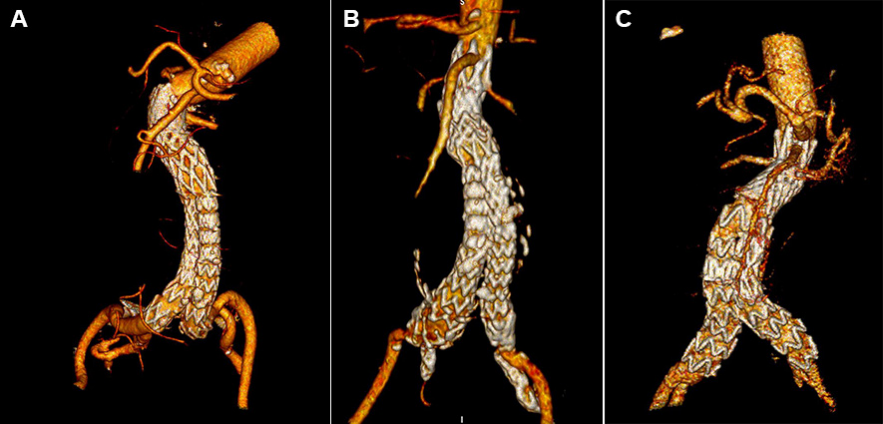
Postoperative 3D CT angiography reconstructions, 30 days after EVAR with the Neoneck technique. (A) 3D CT reconstruction of the first patient treated; (B) 3D CT reconstruction of the second patient treated; (C) 3D CT reconstruction of the third patient treated.

During follow-up with CT angiography (Figures 4-7), an 8% median reduction of the aneurysmal sac was observed on the imaging exams performed after six months of follow-up.

There were no complications related to surgical access and/or deaths registered during this case series.

## DISCUSSION

There is no denying the advances achieved in EVAR over recent years. The improvements in techniques and devices have enabled inclusion of an increasing number of cases that are now treatable with this technique. However, the success of EVAR is dependent on favorable anatomy with an adequate sealing zone. Hostile aortic necks tend to make it difficult to anchor the devices, making type IA endoleaks more likely and increasing the risk of aneurysmal sac rupture and death.^4,8^

In cases of anatomies prohibitive for EVAR, open surgical repair should still be recommended, with excellent results in eligible patients.^4^ However, AAA is very prevalent in elderly patients with comorbidities that may be limiting, such as heart and lung diseases. A significant part of this population may have important risk factors that restrict the choice of open repair due to a high risk of perioperative complications and death.^1,2,4^

The only devices for highly angulated aortic necks (up to 90º) approved for use in Brazil were the Anaconda/Terumo® and the Aorfix/Endovastec®. The first has not been available in Brazil since 2021 and the second is unfortunately not available for use at our service. Recently, Gore® launched endoprostheses capable of accommodating large aortic angles, although the abdominal device is not yet available in Brazil. For this reason, over the years, several techniques have been developed and refined to allow endovascular treatment, even in cases of severe angulation, achieving adequate proximal sealing in EVAR.

In 2013, Chisci et al.^9^ published a case report using the E-XL/Artivion® transrenal stent as a way to treat and prevent type Ia endoleaks in patients with tortuous aortic necks undergoing EVAR. After 15 months’ follow-up there was no evidence of proximal endoleaks. However, it is important to emphasize that the presence of a stent in the transrenal topography can make future approaches more difficult if there is a need to cover visceral arteries and the risks and benefits of its use must therefore be weighed up. In 2017, Takayama et al.^12^ published a proposed technique for angulated necks, using the directional control mechanism of the tip of a C3 Excluder/Gore endoprosthesis® to achieve better fit in the tortuous aortic neck, without evidence of endoleak at 6 months. This method is still experimental, with few reported cases, and no strict definition of which cases are favorable for it and with evident limitations depending on the device used, such as stent grafts with nose cones or suprarenal fixation.

Most of the literature on the Palmaz stent describes its use for correcting type Ia endoleaks in angled aortic necks, after endoprosthesis placement, either at the same surgical intervention or in surgical reapproaches after CT control scans show leakage.^9-11^ There is scant literature on the primary use of stents to remodel the proximal neck and rectify angulation immediately before implantation of the prosthesis. One of the advantages of this preparatory stent placement is to avoid “overballooning” of the proximal neck and to avoid possible dissections and ruptures, since the aortic anatomical configuration has been positively modified, ensuring good circumferential apposition of the prosthesis material to the aortic wall.^9^

Considering the small number of published studies in which the Palmaz stent is previously implanted in the aorta, the main idea of previously implanting the Palmaz stent in an infrarenal position is precisely not to interfere with the fixation of the endoprosthesis, considering that we only use suprarenal fixation devices. With regard to the relationship between the endoprosthesis material and the stent mesh, in this small series we did not identify any interference.

The global literature suggests that the E-XL transrenal stent® (not available in Brazil) is preferred over the Palmaz stent for rectifying the aortic neck, as it is more flexible.^9,10^ This is due to the E-XL’s hybrid nitinol configuration, with an open-cell design in the middle, closed at both ends, constant external radial force along the length of the device. In contrast, the shorter closed cells of the Palmaz stent, made of stainless steel, do not allow such a constant external radial force.^11^ In addition, endoprostheses may continue to expand as the aorta degenerates in one third of patients, generating an enlargement of the neck, with a risk of loss of Palmaz stent apposition to the endoprosthesis, and consequent loss of the proximal sealing zone.^11^ However, as the E-XL stent has a more adequate external radial force, it usually accommodates degenerative neck dilation better, ensuring good apposition and sealing of the graft. The Palmaz stent was deployed in the present study because it is the only option available in Brazil.

Despite being a less invasive treatment than open repair, EVAR employed in cases of unfavorable anatomy, such as hostile aortic necks, generally progresses to a greater need for reinterventions and associated complications.^4^ Use of the Neoneck Palmaz stent technique is presented, both in the literature and in this case series, as a suitable option, with fewer reapproaches in the short and medium term.^9^ However, studies with larger samples and longer follow-up are not yet available to assess the long-term behavior and confirm the most favorable evolution.

The health department where the described treatments were carried out is accredited by the Brazilian Unified Health System (SUS), providing care free of charge, where access to materials is subject to financial limitations. It is often not possible to use the most modern and suitable current devices available or to perform treatments considered gold standard for complex aortic pathologies. In this scenario of limited expenditure, combined with cases of unfavorable aortic angulation and patients with clinical conditions that limit open surgical repair, primary use of the Palmaz stent seems to represent a viable option.

Despite the reduced sample size and brief follow-up time, the data presented in this initial experiment suggest that the “Neoneck” is a promising, effective, technique with a satisfactory rate of technical success (100%), with no evidence of migration and/or type IA endoleak.

## CONCLUSION

This paper presents a series of cases in which the Neoneck technique was feasible and achieved a high rate of technical success, with favorable results in the short and medium term. Prospective studies with long-term clinical follow-up are still needed to fully assess the safety of the technique and validate it.
